# Relapse after withdrawal from anti‐TNF therapy for inflammatory bowel disease: an observational study, plus systematic review and meta‐analysis

**DOI:** 10.1111/apt.13547

**Published:** 2016-02-19

**Authors:** N. A. Kennedy, B. Warner, E. L. Johnston, L. Flanders, P. Hendy, N. S. Ding, R. Harris, A. S. Fadra, C. Basquill, C. A. Lamb, F. L. Cameron, C. D. Murray, M. Parkes, I. Gooding, T. Ahmad, D. R. Gaya, S. Mann, J. O. Lindsay, J. Gordon, J. Satsangi, A. Hart, S. McCartney, P. Irving, UK Anti‐TNF withdrawal study group, C. W. Lees, Tariq Ahmad, Umesh Basavaraju, Christos Christodoulou, Fraser Cummings, Kay Grieveson, Matthew Johnston, Simon Lal, Karen Lithgo, Melanie Lockett, Daniel Maggs, John Mansfield, Joy Mason, Emma Nowell, Miles Parkes, Richard Russell, Abhey Singh, Catherine Stansfield, John Thomson, David C. Wilson

**Affiliations:** ^1^ EdinburghUK; ^2^ LondonUK; ^3^ WinchesterUK; ^4^ Newcastle Upon TyneUK; ^5^ GlasgowUK; ^6^ CambridgeUK; ^7^ ColchesterUK; ^8^ ExeterUK

## Abstract

**Background:**

Infliximab and adalimumab have established roles in inflammatory bowel disease (IBD) therapy. UK regulators mandate reassessment after 12 months' anti‐TNF therapy for IBD, with consideration of treatment withdrawal. There is a need for more data to establish the relapse rates following treatment cessation.

**Aim:**

To establish outcomes following anti‐TNF withdrawal for sustained remission using new data from a large UK cohort, and assimilation of all available literature for systematic review and meta‐analysis.

**Methods:**

A retrospective observational study was performed on 166 patients with IBD (146 with Crohn's disease (CD) and 20 with ulcerative colitis [UC) and IBD unclassified (IBDU)] withdrawn from anti‐TNF for sustained remission. Meta‐analysis was undertaken of all published studies incorporating 11 further cohorts totalling 746 patients (624 CD, 122 UC).

**Results:**

Relapse rates in the UK cohort were 36% by 1 year and 56% by 2 years for CD, and 42% by 1 year and 47% by 2 years for UC/IBDU. Increased relapse risk in CD was associated with age at diagnosis [hazard ratio (HR) 2.78 for age <22 years], white cell count (HR 3.22 for >5.25 × 10^9^/L) and faecal calprotectin (HR 2.95 for >50 μg/g) at drug withdrawal. Neither continued immunomodulators nor endoscopic remission were predictors. In the meta‐analysis, estimated 1‐year relapse rates were 39% and 35% for CD and UC/IBDU respectively. Retreatment with anti‐TNF was successful in 88% for CD and 76% UC/IBDU.

**Conclusions:**

Assimilation of all available data reveals remarkable homogeneity. Approximately one‐third of patients with IBD flare within 12 months of withdrawal of anti‐TNF therapy for sustained remission.

## Introduction

Tumour necrosis factor (TNF) antagonists, notably infliximab (IFX) and adalimumab (ADA) are firmly established induction and maintenance agents in Crohn's disease (CD) and ulcerative colitis (UC).^1^, ^2^, ^3^, ^4^ The European Crohn's and Colitis Organisation (ECCO) recommend their use for CD that is refractory to steroids or relapses after initial therapy, as second‐line therapy in patients with acute severe UC and in patients with immunomodulator‐refractory UC.^5^, ^6^ However, despite the advent of biosimilar infliximab, the drugs are expensive (approximately £6–10 000 per annum)^7^ and there remain some concerns over long‐term safety. Serious potential adverse effects include immunogenicity, opportunistic infections, melanoma.^8^, ^9^ Once sustained deep remission has been achieved on maintenance anti‐TNF therapy clinicians, patients and payers may all have different motivations for a trial of drug withdrawal. Indeed in the UK, the National Institute for Clinical Excellence (NICE) and the Scottish Medicines Consortium (SMC) mandate reassessment at 12 monthly intervals with a consideration of drug cessation where patients are in stable remission. However, there is presently insufficient data on relapse and recapture rates to inform such decision making.^9^, ^10^, ^11^, ^12^ We therefore aimed to examine the rate of disease relapse in IBD patients utilising all available data. We recruited a large retrospective uncontrolled cohort of patients from the UK, all withdrawn from anti‐TNF therapy for sustained clinical remission, and assessed possible predictive factors for relapse and the success of drug reintroduction. We then performed a systematic review of the published literature and conference abstracts with a meta‐analysis of all relevant data.

## Subjects and methods

### Study design

A multi‐centre retrospective clinical audit was conducted using patients identified from 21 IBD centres across the UK. A detailed review of case notes was performed using a standardised proforma and study guide, accessible through the www.ibdscotland.org website. Data were extracted detailing patient demographics including: sex, diagnosis (CD/UC/IBDU), date of and age at diagnosis, weight (at withdrawal) and smoking status. Drug therapy details gathered include: anti‐TNF used, start date, age when started, original approach of therapy, initial and maintenance dosages, stop date, age at withdrawal, tapering at withdrawal and concomitant medication. Parameters at withdrawal included: reason for withdrawal, date of last symptomatic flare and course of systemic corticosteroids prior to withdrawal, Montreal classification and behaviour, laboratory markers [faecal calprotectin, C‐reactive protein (CRP), haemoglobin, platelets, erythrocyte sedimentation rate (ESR), white cell count (WCC), albumin], endoscopic findings and abdominal imaging. Endoscopic findings were given as free text by the individual sites and coded centrally by a single researcher as quiescent mild, moderate or severe. Formal assessment of the endoscopic appearances using a validated score was not deemed feasible. Relapse was also recorded, noting the severity, anti‐TNF reintroduction and need for additional treatment. Eligible patients were identified for the study by searching IBD databases and out‐patient clinic lists at the participating centres.

Patients with IBDU and UC were analysed as a single group since numbers of each individually were small.

### Study criteria

Inclusion criteria were: confirmed diagnosis of IBD, at least 12 months of continuous anti‐TNF therapy, withdrawal for sustained clinical remission and corticosteroid‐free remission for at least 6 months at time of withdrawal. Patients meeting inclusion criteria were identified at each study site, and their suitability for inclusion was checked centrally based on the reported reasons for drug withdrawal and timing of last symptomatic flare, drug withdrawal and follow‐up. Each study site was asked to identify patients by screening all of their patients treated at any time with anti‐TNF in order to reduce bias.

Disease relapse was classified as either moderate or severe. Moderate relapse was defined by the requirement of oral steroids, immunomodulators or recommencement of anti‐TNF therapy. Hospital admission, IV steroids and resectional surgery defined severe relapse.

The pre‐specified primary end‐point was a moderate–severe relapse at 12 months while secondary end‐point was moderate–severe relapse at 24 months.

### Statistical analysis

Data were collected by each site in a Microsoft Excel spreadsheet (Microsoft Corp, Redmond, WA, USA) and submitted to the lead site. Anonymised data were then collated in a single master spreadsheet. Each entry was rechecked to make sure they met the inclusion criteria. Data were analysed using R 3.1.3 (R Foundation for Statistical Computing, Vienna, Austria). Survival analysis including Cox proportional hazards and Kaplan–Meier analysis were done using the *survival* package.^13^, ^14^ The overall moderate‐to‐severe relapse rates were estimated using the Kaplan–Meier method. These were divided into moderate and severe relapse based on the proportions of relapses of each category by that time point.

For Cox proportional hazards analysis, continuous variables were analysed using untransformed values for those with approximately normal distributions, and log‐transformed values for those with approximately log‐normal distributions (CRP and faecal calprotectin). For univariable analysis of variables with missing data, only individuals with known data were included. Colonoscopies were categorised as quiescent, mild and moderate inflammation, and for statistical analysis were split into no inflammation vs. a mild or greater degree of inflammation. Blood tests were only analysed for those patients without an additional reason for anti‐TNF withdrawal which might have influenced the results. Continuous variables that were significant on univariable analysis were also analysed as a categorical variable using a threshold derived that gave the highest sum of sensitivity and specificity for predicting relapse at 12 months. Multivariable analysis was performed on variables with *P* < 0.1 on univariable analysis and with at least 100 individuals with data. After creating an initial model, backwards step‐wise regression was performed using the Akaike An Information Criterion (AIC) to select which variables to keep. A second model was created which also included faecal calprotectin, since it was one of the most significant and clinically relevant markers; this could only include the subset of patients with faecal calprotectin results.

### Systematic review

#### Criteria for including studies

##### Types of studies

Retrospective or prospective uncontrolled or controlled studies.

##### Types of participants

Patients with IBD withdrawn from anti‐TNF therapy after a period of sustained clinical remission (at least 6 months).

##### Types of interventions

Withdrawal of anti‐TNF therapy.

##### Types of outcome measures

Proportion of patients experiencing clinical relapse by 1 year following treatment withdrawal.

##### Exclusion criteria

Studies without an estimate of 1‐year relapse; studies where the outcome measure was endoscopic recurrence rather than clinical relapse; studies where anti‐TNF was being used as post‐operative prevention of recurrence.

#### Search methods for identification of studies

Computer‐assisted searches of PubMed and EMBASE were carried out covering the years 1950–2015 (PubMed) and 1980–2015 (EMBASE). PubMed search terms used were: Search (anti‐TNFa OR antiTNF OR antiTNFa OR “anti‐tumour necrosis” OR “anti‐tumor necrosis” OR infliximab OR adalimumab OR anti‐TNF OR golimumab OR certolizumab) AND (withdrawal OR discontinuation OR cessation OR stopping OR de‐escalation) AND (inflammatory bowel disease OR IBD OR Crohn's OR colitis OR Crohn). EMBASE was searched using the same strategy, but combining three searches together with the AND operator. No limits were imposed on either type of search, and searches were last updated on 6 March 2015. Where available, EMBASE search results were assigned PubMed IDs using the PubMed batch citation tool. The results of all searches were then merged with those from PubMed and duplicates removed by matching PubMed IDs and manual matching of titles/journals. Where an identical abstract had been presented at two or more conferences, these were also regarded as duplicates. EMBASE includes conferences from 2009 onwards.

##### Data collection and analysis

All titles identified by the above searches were reviewed. Abstracts and full texts of relevant papers that related to withdrawal of anti‐TNF in IBD were reviewed to identify independent data sets that met the inclusion criteria. Data were extracted and stored in an Excel spreadsheet by a single researcher (NAK). Studies were assessed as to whether they were prospective or retrospective, controlled or uncontrolled. Data extracted included an estimate of the 12‐month relapse rate (controlling for loss‐to‐follow‐up where relevant) and variables predictive of relapse.

### Meta‐analysis

Meta‐analysis was performed using the metafor package in R 3.2.2.^15^ Proportions were used as the measure of effect size and a random effects model to estimate the average proportion. Weighting was done with the inverse variance method. Proportion data were transformed using the arcsine square root transformation and reverse transformed for display. 0.5 was added to each count where there was a zero (e.g. in the situation of 100% success with retreatment). Heterogeneity was estimated using the restricted maximum‐likelihood estimator. An *I* ^2^ of less than 40% was regarded as likely to be unimportant.^16^ A *P* value for heterogeneity was also calculated using Cochran's Q method.^17^ Publication bias was assessed using a funnel plot. The primary analysis was performed using studies that included patients with at least 12 months' therapy. A further analysis was performed also including studies with a shorter minimum time on anti‐TNF, though still only examining studies where patients were withdrawn from maintenance therapy.

## Results

### Retrospective UK cohort

Out of the 21 centres across the UK, 166 patients, 146 with CD and 20 with UC/IBDU, were eligible for inclusion (Table 1). A further 19 patients were submitted but excluded from analysis, most commonly for less than 12 months' therapy or missing data. The number of screened patients was not available across most sites, but for Edinburgh 380 patients were screened to identify 10 that met the inclusion criteria with the majority of the remainder either continuing with anti‐TNF (*n* = 147) or having been withdrawn for reasons other than sustained remission (*n* = 155).

**Table 1:** Demographics of patients in the UK retrospective study

	Crohn's disease (*n* = 146)	Ulcerative colitis/IBDU (*n* = 20)
Anti‐TNF used		
Infliximab	117 (80%)	19 (95%)
Adalimumab	29 (20%)	1 (5%)
Sex		
Female	83 (57%)	8 (40%)
Age at anti‐TNF withdrawal/years	31 (24–42)	40 (29–46)
Reason for starting anti‐TNF		
Failure of immunomodulators	117/139 (84%)	14/18 (78%)
Early combination therapy	7/139 (5%)	0
Early monotherapy	3/139 (2%)	0
Hospitalisation for acute severe disease	5/139 (4%)	4/18 (22%)
Other	7/139 (5%)	0
Time on anti‐TNF/months	29 (18–45)	21 (14–33)
Follow‐up time since withdrawal/months	24 (15–38)	23 (15–35)
Year stopped anti‐TNF	2012 (2010–2012)	2012 (2011–2013)
Smoking at withdrawal		
Current	14/129 (11%)	1/17 (6%)
Ex	18/129 (14%)	3/17(18%)
Never	97/129 (75%)	13/17 (76%)
Montreal location		
L1 ± L4	18/142 (13%)	
L2 ± L4	38/142 (27%)	
L3 ± L4	81/142 (57%)	
L4	5/142 (4%)	
Montreal behaviour		
B1	98/142 (69%)	
B2	21/142 (15%)	
B3	23/142 (16%)	
Montreal extent		
E2		9/19 (47%)
E3		10/19 (53%)
Previous surgical resection for IBD	35/125 (28%)	0/17 (0%)
Therapy at withdrawal		
Azathioprine	66/146 (45%)	12/20 (60%)
Mercaptopurine	9/145 (6%)	1/20 (5%)
Methotrexate	20/145 (14%)	2/20 (10%)
Mesalazine	17/146 (12%)	7/20 (35%)
Any of the above	107/145 (73%)	16/20 (80%)

Numbers shown are medians and interquartile ranges or numbers and percentages as appropriate. Percentages have been calculated after exclusion of missing data within each category.

One hundred and seventeen (80%) CD patients and 19 (95%) UC/IBDU patients were on infliximab prior to withdrawal; the remainder were on adalimumab. The median time taken for introducing anti‐TNF therapy post‐diagnosis was 63 months for CD [interquartile range (IQR) 30–122] and 22 months for UC/IBDU (IQR 10–70). Median therapy duration prior to withdrawal was 29 months (IQR 18–45) for CD and 21 months for UC/IBDU (IQR 14–33). Median follow‐up was 24 months (IQR 15–38) for CD and 23 months (IQR 15–35) for UC/IBDU. Investigations at withdrawal are shown in Table 2.

**Table 2:** Investigations at withdrawal of anti‐TNF in the UK retrospective study

	Crohn's disease		Ulcerative colitis/IBDU	
	*n*	Median (IQR) or *n* (%)	*n*	Median (IQR) or *n* (%)
Haemoglobin (g/L)	133	137 (128–146)	20	132 (126–142)
White cell count (10^9^/L)	133	6.2 (5.0–7.4)	20	6.6 (5.4–8.0)
Platelet count (10^9^/L)	133	256 (213–299)	20	260 (216–351)
Albumin (g/L)	128	44 (40–46)	19	39 (37–44)
CRP (mg/L)	129	2.5 (1.5–3.0)	18	2.2 (1.5–4.5)
Faecal calprotectin (μg/g)	46	46 (20–91)	3	<20 (<20–334)
Colonoscopy				
Quiescent	84	74 (88%)	16	12 (75%)
Mild		9 (11%)		2 (12%)
Moderate		1 (1%)		2 (12%)

For all blood tests, patients were only included in this analysis if they had no additional reasons for anti‐TNF withdrawal (*n* = 138 for Crohn's disease and 20 for ulcerative colitis/IBDU). No full blood count was performed at withdrawal on five CD patients (three of whom were children). Colonoscopy was performed on 84 of the Crohn's disease patients and 16 of the ulcerative colits/IBDU patients.

The majority of patients in both disease groups (80% CD, 78% UC/IBDU) commenced anti‐TNF following failure of immunomodulators. Among the CD cohort, 69% had inflammatory (Montreal B1) disease, with the remainder split between stricturing (B2) and penetrating (B3). While all patients had to be in clinical remission for 6 months at the point of treatment withdrawal, there was an additional factor that influenced the decision for withdrawal in 21 (14%) CD patients and 1 (5%) UC/IBDU patient, including planned pregnancy or mild drug intolerance (Table S1).

### Relapse rate and predictive factors

**Figure 1:**
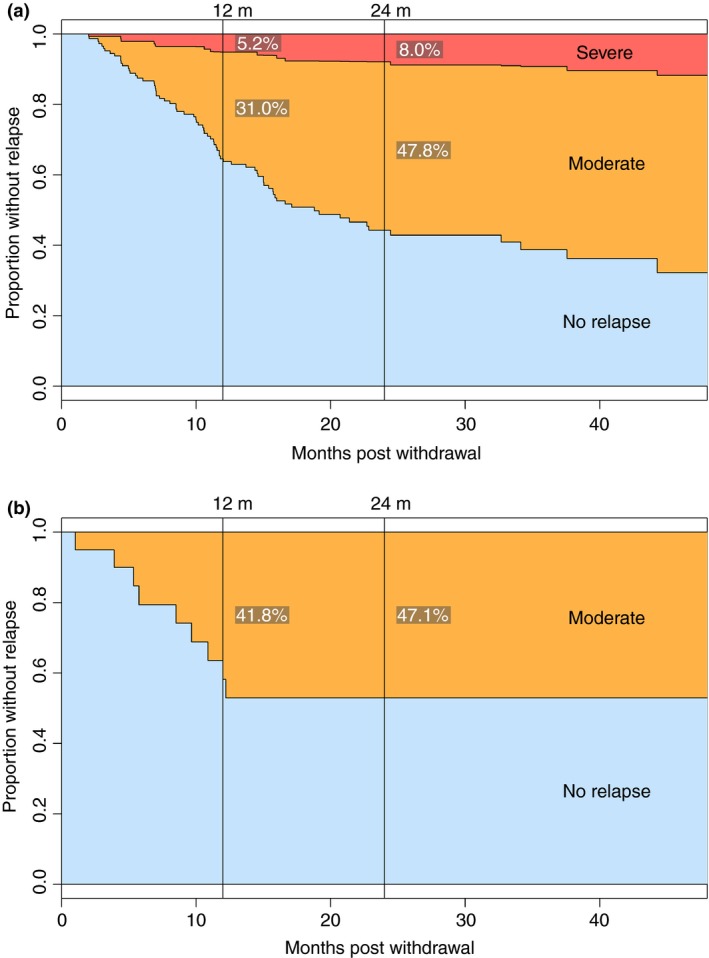
Survival analysis of relapse following withdrawal of anti‐TNF for sustained remission of Crohn's disease (a) and ulcerative colitis/IBD unclassified (b) in the UK retrospective study.

By time of last follow‐up, 75/146 (51%) CD patients and 9/20 (45%) UC/IBDU patients had experienced relapse (Figure 1). By 12 months, the estimated moderate‐to‐severe relapse rate was 36% in CD [95% confidence interval (CI) 29–44] and 42% in UC/IBDU (95% CI 15–60) at 12 months. By 24 months, the estimated relapse rates had increased to 56% in CD (95% CI 46–64) and 47.1% in UC/IBDU (95% CI 19–65). There was no significant difference in relapse rates between CD and UC/IBDU (*P* = 0.95).

**Figure 2:**
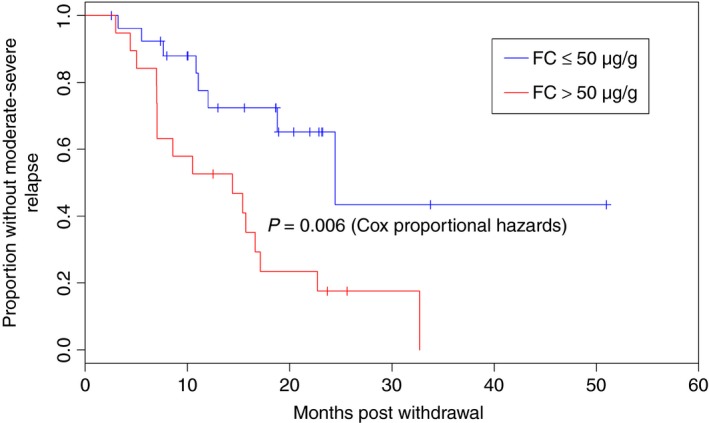
Relapse in Crohn's disease patients following withdrawal of anti‐TNF stratified by faecal calprotectin (FC) (*n* = 46) in the UK retrospective study.

**Figure 3:**
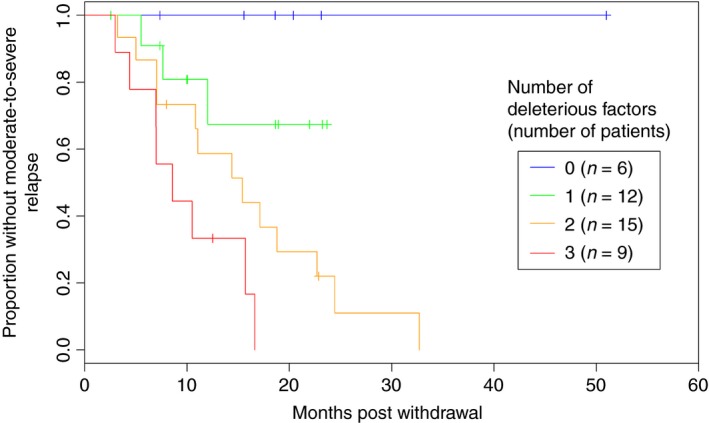
Relapse in Crohn's disease patients following withdrawal of anti‐TNF stratified by faecal calprotectin, white cell count and age at diagnosis in the UK retrospective study.

Predictive factors assessed for relapse are shown in Table 3. Relapse in CD was associated with younger age at diagnosis (*P* = 0.007), white cell count at time of anti‐TNF withdrawal (*P* = 0.013), isolated L4 disease (*P* = 0.005), absence of perianal disease (*P* = 0.045), Montreal behaviour B2 (*P* = 0.024) and log (faecal calprotectin) (*P* = 0.041). Stratifying patients into groups based on faecal calprotectin with a cut‐off of 50 μg/g showed clear separation of the survival curves, with *P* = 0.006 (Figure 2). On multivariable analysis of variables with univariable *P* < 0.1 and *n* > 100 (Table 3b), age at diagnosis (*P* = 0.002) and white cell count >5.25 × 10^9^ (*P* = 0.022) remained significant. This analysis included the 128 patients with data for all included variables faecal calprotectin >50 μg/g was also significant when included in a multivariable model (*P* = 0.016), though this reduced the number of assessable patients to 42. A score comprised of white cell count, age at diagnosis and faecal calprotectin using the thresholds described above showed significant separation of survival curves (*P* < 0.001, Figure 3).

**Table 3:** Predictive factors for relapse after withdrawal from anti‐TNF in Crohn's disease using Cox proportional hazards model in the UK retrospective study. (a) univariable analysis; (b) multivariable analysis

(a)			
	n	HR (95% CI)	*P*
Sex			
Male	146	1.22 (0.77–1.93)	0.389
Smoking at withdrawal			
Never	129	Reference	
Current		1.29 (0.65–2.56)	0.459
Ex		0.72 (0.32–1.59)	0.416
Age at diagnosis (years)	**144**	**0.97 (0.94–0.99)**	**0.007**
Age at diagnosis < 22 years	**144**	**2.71 (1.66–4.43)**	**<0.0001**
Age when starting anti‐TNF (years)	**145**	**0.98 (0.96–1.00)**	**0.046**
Additional reason for anti‐TNF withdrawal	146	0.66 (0.32–1.38)	0.270
Tapered at withdrawal	145	1.02 (0.37–2.79)	0.975
Montreal location			
L1	142	Reference	
L2		1.82 (0.72–4.58)	0.203
L3		2.05 (0.87–4.84)	0.100
**L4**		**5.43 (1.65–17.93)**	**0.005**
Montreal behaviour			
B1	142	Reference	
**B2**		**1.93 (1.09–3.40)**	**0.024**
B3		0.52 (0.24–1.09)	0.084
Perianal disease	**142**	**0.54 (0.30–0.99)**	**0.045**
Immunomodulator at withdrawal	146	0.68 (0.43–1.08)	0.101
Immunomodulator or 5ASA at withdrawal	146	0.77 (0.47–1.28)	0.316
Previous surgical resection	125	1.44 (0.86–2.39)	0.163
Haemoglobin (g/L)	133	1.01 (0.99–1.04)	0.147
White cell count (10^9^/L)	**133**	**1.16 (1.03–1.30)**	**0.013**
White cell count >5.25 × 10^9^/L	**133**	**2.54 (1.39–4.66)**	**0.003**
Platelet count (10^9^/L)	133	1.00 (1.00–1.01)	0.326
CRP [log_10_ (mg/L)]	129	0.83 (0.44–1.55)	0.557
Albumin (g/L)	128	1.00 (0.94–1.05)	0.891
Faecal calprotectin >50 μg/g	**46**	**3.32 (1.42–7.79)**	**0.006**
Faecal calprotectin [log_10_ (μg/g)]	**46**	**1.82 (1.03–2.82)**	**0.041**
Inflammation at colonoscopy	84	0.93 (0.39–2.20)	0.863

(b)				
	Model without calprotectin (*n* = 128)		Model with calprotectin (*n* = 42)	
	HR (95% CI)	*P*	HR (95% CI)	*P*
Age at diagnosis <22 years	**2.29 (1.35–3.88)**	**0.002**	**2.78 (1.11–7.00)**	**0.03**
Montreal behaviour				
B1	Reference			
B2	1.60 (0.88–2.90)	0.200		
B3	0.51 (0.24–1.09)	0.089		
White cell count >5.25 × 10^9^/L	**2.06 (1.11–3.80)**	**0.022**	**3.22 (0.95–10.93)**	**0.06**
Faecal calprotectin >50 μg/g			**2.95 (1.22–7.12)**	**0.02**

HR, hazard ratio; CI, confidence interval.

*P* values less than 0.05 are highlighted in bold. For continuous variables, hazard ratios shown are for each unit increase for age, haemoglobin, white cell count, platelet count and albumin. For CRP and calprotectin which have a log‐normal distribution, hazard ratios shown are for each 10‐fold increase.

There were no associations with any predictive factors for UC/IBDU.

### Consequences of relapse and re‐treatment

Among the 48 CD patients who relapsed in the first 12 months, 22 (46%) required systemic corticosteroid therapy, 7 (15%) required hospital admission and 1 (2%) underwent surgery. Among UC/IBDU patients relapsing in the first 12 months, four (50%) required systemic corticosteroids and 1 (12%) underwent colectomy.

### Reintroduction of anti‐TNF therapy

Anti‐TNF therapy was reintroduced in 56/75 (75%) CD patients and 3/9 (33%) UC/IBDU patients with relapse. The same anti‐TNF was reintroduced in 47/56 (84%) CD patients and 3/3 (100%) UC/IBDU patients, with the remainder switching from infliximab to adalimumab. Reintroduction was deemed successful in 52/56 (93%) with CD and 2/3 (67%) with UC/IBDU. However, in 21 of these 52 CD patients (40%) systemic steroids were also required, and in 2/52 (4%) resectional surgery was needed. None of the three UC/IBDU patients in whom anti‐TNF was introduced required surgery, though the unsuccessful UC/IBDU patient required systemic steroids.

### Systematic review

**Figure 4:**
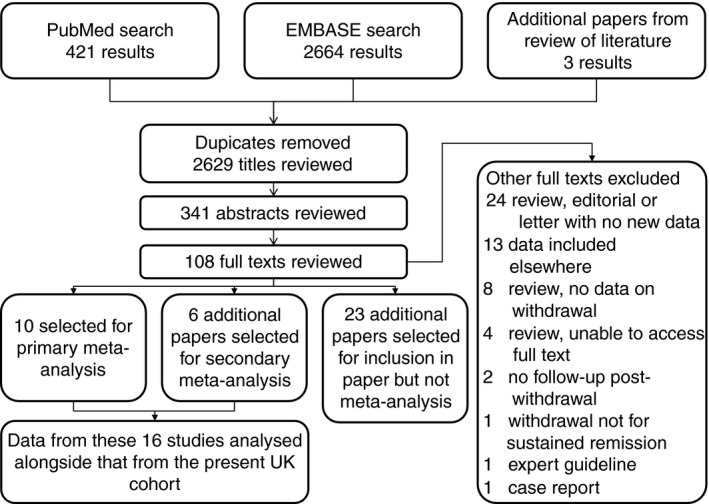
Inclusion flowchart for systematic review/meta‐analysis.

Initial searches and review of bibliographies identified 2629 papers after removal of duplicates (Figure 4). Sixteen studies were deemed eligible for inclusion in the meta‐analysis in addition to the present study. Overall, 12 studies covered CD only, 1 UC only and 4 both diseases (Table S2, excluded studies in Table S3). All of the included studies were uncontrolled observational studies, with a mixture of prospective and retrospective approaches. Twelve studies met the inclusion criteria for the primary meta‐analysis (at least 12 months' anti‐TNF therapy prior to withdrawal).

### Meta‐analysis

**Figure 5:**
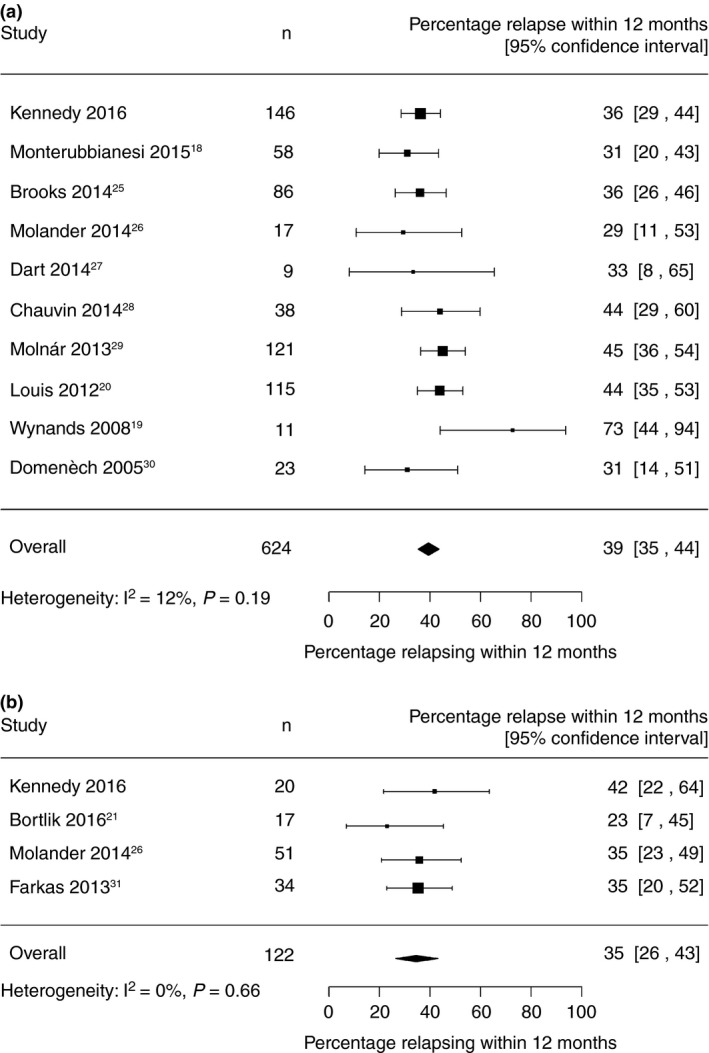
Forest plot for relapse by 12 months after anti‐TNF withdrawal for CD (a) and UC/IBDU (b).

In the primary meta‐analyses, 624 CD patients from 10 studies and 122 UC/IBDU patients from 4 studies were included. The estimated average 12‐month relapse rate was 39% for CD (95% CI 35–44) and 35% for UC/IBDU (95% CI 26–43) (Figure 5). Both data sets had low heterogeneity, with *I* ^2^ = 12% for CD and 0% for UC/IBDU. Expanding the inclusion criteria to include patients with shorter periods on anti‐TNF prior to drug withdrawal increased the heterogeneity to *I* ^2^ = 40% for CD and 56% for UC (Figure S1).

For CD, the estimated average 24‐month relapse rate was 54% (95% CI 49–59) when using the four studies with relevant data that met the primary inclusion criteria (Figure S2A). Extending this to all eight studies with available data gave a similar estimated average relapse rate of 53% (95% CI 49–57) (Figure S3). For UC, there were only two studies with 24‐month relapse date. The estimated average relapse rate was 42% (95% CI 27–58) (Figure S2B).

The estimated average rate of success of retreatment was 88% for CD (95% CI 78–95) and 76% for UC/IBDU (95% CI 56–92) (Figure S4). For CD, there was significant heterogeneity of retreatment success (*I* ^2^ = 73%, *P* < 0.01), though this disappeared when the Monterubbianesi^18^ study was removed (*I* ^2^ without Monterubbianesi study 38%, *P* = 0.21). For UC, the total numbers were low (28 individuals across three studies) and so the confidence intervals were wide.

A funnel plot to assess for publication bias was symmetric for CD (Figure S5), though there was a single outlier (the paediatric Wynands *et al*. study^19^). There were too few points to make a meaningful assessment of publication bias for UC/IBDU.

## Discussion

In patients with IBD withdrawn from anti‐TNF therapy while in sustained remission, our meta‐analysis has shown a clinical relapse rate by 12 months of 39% for CD and 37% for UC/IBDU. For CD, the 24‐month relapse rate was 54%. These estimates are based on our large UK retrospective uncontrolled cohort and assimilation of all presently available and relevant data from the literature. The meta‐analysis reported herein is remarkable for the lack of heterogeneity among the individual data sets. With the important caveat that relatively fewer UC patients were available for analysis, the relapse rates are broadly similar to those observed in CD.

These data will give confidence to clinicians when discussing with patients established on anti‐TNF therapy the chances of disease flare if the drugs are withdrawn. Approximately one in three patients with any form of inflammatory bowel disease are likely to experience a moderate‐to‐severe flare within 12 months of drug withdrawal, and one in two by 24 months. These odds are likely to seem unfavourable to many clinicians and patients but should prove useful when set alongside other key factors. These might include how the timing of drug withdrawal fits in with a patient's life (e.g. important education, work, or family events). In some countries, such as parts of the UK, drug withdrawal will be recommended by regulatory authorities.

Two other key pieces of information are likely to be useful to fully inform clinical teams about making key alterations in drug therapy. Firstly, if a patient does experience a disease flare following drug withdrawal, what are the consequences of this and how successful is reintroduction of drug therapy? Our large UK cohort offers some useful guidance on this. While just under one half of all patients who relapsed required systemic corticosteroids, hospitalisation rates were relatively low (17% in CD, zero in UC) and surgical rescue was a rare event (only two patients). When anti‐TNF therapy was restarted it was deemed to be successful in over 90% of patients with CD. These results were confirmed in the meta‐analysis, with an estimated retreatment success rate of 88% (95% CI 78–95). There was a single outlying study (Monterubbianesi^18^); the reasons for this are unclear, particularly since this study has only been published as a conference abstract, but may reflect different criteria for retreatment success. We have insufficient data to draw any meaningful conclusions in UC.

Secondly, what are the predictive factors of relapse at the time of drug withdrawal? Arguably this is the most important piece of information to enable rationale stratification of patients at drug withdrawal. In our cohort on multivariable analysis, younger age at diagnosis, white cell count and faecal calprotectin were predictive of relapse at 12 months in CD. In contrast, evidence of disease activity at colonoscopy was not predictive, though only 12% of those colonoscoped had any evidence of disease activity. In fact, of the six factors identified in the simplified model of the STORI study, only faecal calprotectin and white cell count were significant in the present study.^20^ L4 disease was also predictive, but the generalisability of this finding is limited by the small numbers in that disease group (five patients).

At a fundamental level we still cannot address the question of whether evidence of active disease at drug withdrawal predicts disease relapse. Central to this paradox is the question whether patients on anti‐TNF therapy with complete mucosal healing are in complete remission because of their ongoing therapy or in spite of it. One might expect those patients who exhibit ongoing mucosal inflammation despite being in complete clinical remission to be at much higher risk of disease relapse following drug withdrawal. In our study, a faecal calprotectin above 50 μg/g was predictive in the Crohn's cohort of relapse (HR 3.32, *P* = 0.006). faecal calprotectin was also found to be predictive in the STORI study,^20^ while Bortlik *et al*. found no association of faecal calprotectin with relapse.^21^ Inconsistencies across the literature in large part reflect that in most cohorts very few patients had any objective evidence of mucosal inflammation at drug withdrawal. The data from our study are typical: 83% of patients had a normal CRP and 88% a calprotectin <250 μg/g with 60% having calprotectin <50 μg/g. Although white cell count was an independent predictive factor in this cohort, the optimum threshold was well within the normal range at 5.25 × 10^9^/L (and was not related to immunomodulator use). In addition, full colonoscopic assessment, where it was performed, was completely normal in 86/100. This is despite clinical remission at the time of drug withdrawal being the only major inclusion criterion in our protocol. Clinicians are evidently continuing therapy in patients where there is evidence of ongoing inflammation, often in spite of drug optimisation. For many, this will reflect a lack of alternative therapeutic options. However, there are likely to be a substantial number of patients in whom anti‐TNF therapy can be discontinued in this scenario as it is having no discernible impact on the disease course. This is highlighted by results from the STORI trial where undetectable infliximab trough levels were associated with a lower risk of subsequent relapse on drug withdrawal.^20^ Further prospective clinical trials are planned to address these key issues.

The limitations of our UK cohort are its retrospective nature, and the missing data at the time of drug withdrawal. CRP, calprotectin and colonoscopic assessment were available in 93%, 30% and 60% of patients. The relatively low number of abnormal colonoscopies may have limited the power of the study for this predictive marker. Details of small bowel imaging were only available in 30 patients, and the data were too heterogeneous (ultrasound, CT, MRI, barium studies) to allow meaningful analysis. All sites were asked to be thorough in their searches of anti‐TNF‐treated patients to reduce the risk of selection bias, but this remains a potential concern. In Edinburgh, where the lead authors are based, it should be noted that even with comprehensive review of our patient cohort, it was in practice very difficult to identify patients who met our criteria. This suggests that despite NICE guidance, relatively few patients with IBD have their anti‐TNF therapy stopped for sustained remission. It is also of note that 75% of CD patients in the UK cohort were never smokers, which is lower than reported elsewhere.^22^, ^23^, ^24^ This may reflect clinician concerns about relapse rates in smokers whose anti‐TNF therapy is withdrawn.

We have been thorough in our systematic review and followed strict criteria and guidelines for the selection of studies for the meta‐analysis. However, all of the included studies were uncontrolled and many were retrospective. It is therefore not possible to draw conclusions about what would have happened in the absence of drug withdrawal. For the retrospective cohorts in particular, there is a risk of recall bias, which could inflate or reduce estimates of relapse rate in those cohorts; however, there was no significant difference in the 12‐month relapse rate between prospective and retrospective cohorts.

We are, however, able to draw several important conclusions based on a synthesis of all available data. Approximately one‐third of patients with CD or UC in sustained clinical remission are likely to suffer a disease relapse within 12 months of planned drug withdrawal. We are presently unable to predict which patients are most likely to flare in this situation. We can recapture disease remission by restarting anti‐TNF therapy in the majority of patients, although nearly half may also require a course of corticosteroids. Clinicians and patients should weigh up the decisions about drug withdrawal on an individual basis taking into account the preceding disease course, and the appropriate time in a patient's life for such critical therapeutic changes.

## Authorship

*Guarantor of the article*: Dr Charles W. Lees.

*Author contributions*: NAK and CWL contributed towards study design; NAK, BW, ELJ, LF, PH, NSD, RH, ASF, CB, CAL, FLC, DG, with supervision from CDM, MP, IG, TA, SM, JL, JG, JS, AH, SM, PMI and CWL contributed towards data collection; NAK contributed towards data analysis; CB, NAK and CWL contributed towards the writing of the paper with subsequent review and input from the other authors.

All authors approved the final version of the manuscript.

## Supporting information


**Table S1.** Detailed reasons for withdrawal from anti‐TNF.
**Table S2.** Characteristics of articles included in systematic review and meta‐analysis of withdrawal of anti‐TNF for sustained clinical remission, sorted by year of publication. (A) Studies with at least 1 year anti‐TNF prior to withdrawal. (B) Studies with maintenance therapy but <1 year anti‐TNF at withdrawal. C: Studies included in secondary meta‐analysis but excluded from primary meta‐analysis for other reasons.
**Table S3.** Studies excluded from meta‐analysis.
**Figure S1.** Forest plot for relapse by 12 months after anti‐TNF withdrawal for CD (A) and UC/IBDU (B) including all studies with patients treated with maintenance anti‐TNF.
**Figure S2.** Forest plot for relapse by 24 months after anti‐TNF withdrawal for CD (A) and UC/IBDU (B) including only studies with patients treated with maintenance anti‐TNF for at least 12 months.
**Figure S3.** Forest plot for relapse by 24 months after anti‐TNF withdrawal for CD including all studies of patients treated with maintenance anti‐TNF.
**Figure S4.** Forest plot for success rates of reintroduction of anti‐TNF after withdrawal for CD (A) and UC/IBDU (B) including all studies with patients treated with maintenance anti‐TNF.
**Figure S5.** Funnel plot for relapse by 12 months after anti‐TNF withdrawal including studies with patients treated with maintenance anti‐TNF for at least one year prior to withdrawal.

## Appendix 1

### Full list of contributors to ‘Outcomes after withdrawal from anti‐TNF therapy for inflammatory bowel disease: an observational study, systematic review and meta‐analysis'

Tariq Ahmad^13^, Umesh Basavaraju^16^, Catriona Basquill^1^, Fiona L. Cameron^8,9^, Christos Christodoulou^11^, Fraser Cummings^17^, Nik S. Ding^4^, Adam S. Fadra^6^, Lucy Flanders^3^, Daniel R. Gaya^14^, Ian Gooding^12^, John Gordon^5^, Kay Grieveson ^10^, Richard Harris^5^, Ailsa Hart^4^, Philip Hendy^4^, Peter Irving^2^, Emma L. Johnston^2^, Matthew Johnston^18^, Nicholas A. Kennedy^1^, Simon Lal^19^, Christopher A. Lamb^7^, Charlie W. Lees^1^, James O. Lindsay^6^, Karen Lithgo^18^, Melanie Lockett^20^, Daniel Maggs^20^, Steve Mann^15^, John Mansfield^21^, Joy Mason^12^, Sara McCartney^3^, Charles D. Murray^10^, Emma Nowell^22^, Miles Parkes^11^, Richard Russell^9^, Jack Satsangi^1^, Abhey Singh^13^, Catherine Stansfield^19^, John Thomson^16^, Ben Warner^2^, David C. Wilson^8^

^1^Gastrointestinal Unit, Western General Hospital, Edinburgh, UK. ^2^Gastroenterology, Guy's & St Thomas' NHS Foundation Trust, London, UK. ^3^Department of Gastroenterology, University College London Hospitals NHS Foundation Trust, London, UK. ^4^Inflammatory Bowel Disease Unit, St Mark's Hospital, London, UK. ^5^Department of Gastroenterology, Royal Hampshire County Hospital, Hampshire Hospitals NHS Foundation Trust, Winchester, UK. ^6^Department of Gastroenterology, The Royal London Hospital, Barts Health NHS Trust, London, UK. ^7^Institute of Cellular Medicine, Newcastle University, Newcastle Upon Tyne, UK. ^8^Paediatric Gastroenterology, Royal Hospital for Sick Children, Glasgow, UK. ^9^Paediatric Gastroenterology and Nutrition, Child Life and Health, University of Edinburgh, Edinburgh, UK. ^10^Department of Gastroenterology, Royal Free Hospital, Royal Free London NHS Foundation Trust, London, UK. ^11^Department of Gastroenterology, Addenbrooke's Hospital, Cambridge, UK. ^12^Department of Gastroenterology, Colchester Hospital University NHS Foundation Trust, Colchester, UK. ^13^Department of Gastroenterology, Royal Devon and Exeter NHS Foundation Trust, Exeter, UK. ^14^Department of Gastroenterology, Glasgow Royal Infirmary, Glasgow, UK. ^15^Department of Gastroenterology, Barnet & Chase Farm Hospitals, Royal Free London NHS Foundation Trust, London, UK. ^16^Department of Gastroenterology, Aberdeen Royal Infirmary, Aberdeen, UK. ^17^Department of Gastroenterology, University Hospital Southampton, Southampton, UK. ^18^Department of Gastroenterology, Luton & Dunstable University Hospital foundation trust NHS, Luton, UK. ^19^Department of Gastroenterology, Salford Royal NHS Foundation Trust, Salford, UK. ^20^Department of Gastroenterology, North Bristol NHS Trust, Bristol, UK. ^21^Department of Gastroenterology, Royal Victoria Infirmary, Newcastle upon Tyne, UK. ^22^Department of Gastroenterology, Hairmyres Hospital, East Kilbride, UK.
