# Supplementary material for: Relapse after withdrawal from anti‐TNF therapy for inflammatory bowel disease: an observational study, plus systematic review and meta‐analysis
Source: Aliment Pharmacol Ther. 2016 Feb 19;43(8):910–23. doi: 10.1111/apt.13547 (PMC4793922; doi:10.1111/apt.13547)

Full list of contributors to 'Outcomes after withdrawal from anti-TNF therapy for inflammatory bowel disease: an observational study, systematic review and meta-analysis'

Tariq Ahmad[13], Umesh Basavaraju[17], Catriona Basquill[1], Catriona Basquill[1], Fiona Cameron[8,9], Christos Christodoulou[11], Fraser Cummings[18], Nik Ding[4], Nik Ding[4], Adam S Fadra[6], Lucy Flanders[3], Daniel Gaya[14], Ian Gooding[12], John Gordon[5], Kay Grieveson [10], Richard Harris[5], Ailsa Hart[4], Philip Hendy[4], Peter Irving[2], Emma Johnston[2], Matthew Johnston[19], Nicholas A Kennedy[1], Simon Lal[20], Christopher A Lamb[7], Charlie W Lees[1], James Lindsay[6], Karen Lithgo[19], Melanie Lockett[21], Daniel Maggs[21], Steve Mann[15], John Mansfield[22], Joy Mason[12], Sara McCartney[3], Charles Murray[10], Emma Nowell[23], Miles Parkes[11], Richard Russell[9], Jack Satsangi[1], Abhey Singh[13], Catherine Stansfield[20], John Thomson[17], Ben Warner[2], David C Wilson[8]

[1]Gastrointestinal Unit, Western General Hospital, Edinburgh, UK. [2]Gastroenterology, Guy's & St Thomas' NHS Foundation Trust, London, UK. [3]Department of Gastroenterology, University College London Hospitals NHS Foundation Trust, London, UK. [4]Inflammatory Bowel Disease Unit, St Mark's Hospital, London, UK. [5]Department of Gastroenterology, Royal Hampshire County Hospital, Hampshire Hospitals NHS Foundation Trust, Winchester, UK. [6]Department of Gastroenterology, The Royal London Hospital, Barts Health NHS Trust, London, UK. [7]Institute of Cellular Medicine, Newcastle University, Newcastle Upon Tyne, UK. [8]Paediatric Gastroenterology, Royal Hospital for Sick Children, Glasgow, UK. [9]Paediatric Gastroenterology and Nutrition, Child Life and Health, University of Edinburgh, Edinburgh, UK. [10]Dept of Gastroenterology, Royal Free Hospital, Royal Free London NHS Foundation Trust, London, UK. [11]Dept of Gastroenterology, Addenbrooke's Hospital, Cambridge, UK. [12]Dept of Gastroenterology, Colchester Hospital University NHS Foundation Trust, Colchester, UK. [13]Dept of Gastroenterology, Royal Devon and Exeter NHS Foundation Trust, Exeter, UK. [14]Dept of Gastroenterology, Glasgow Royal Infirmary, Glasgow, UK. [15]Dept of Gastroenterology, Barnet & Chase Farm Hospitals, Royal Free London NHS Foundation Trust, London, UK. [16]A list of members and affiliations appears in the Supplementary Note. [17]Dept of Gastroenterology, Aberdeen Royal Infirmary, Aberdeen, UK. [18]Dept of Gastroenterology, University Hospital Southampton, Southampton, UK. [19]Dept of Gastroenterology, Luton & Dunstable University Hospital foundation trust NHS, Luton, UK. [20]Dept of Gastroenterology, Salford Royal NHS Foundation Trust, Salford, UK. [21]Dept of Gastroenterology, North Bristol NHS Trust, Bristol, UK. [22]Dept of Gastroenterology, Royal Victoria Infirmary, Newcastle upon Tyne, UK. [23]Dept of Gastroenterology, Hairmyres Hospital, East Kilbride, UK.

Supplementary tables for 'Outcomes after withdrawal from anti-TNF therapy for inflammatory bowel disease: an observational study, systematic review and meta-analysis'

Supplementary table 1 – Detailed reasons for withdrawal from anti-TNF

Reason for withdrawal		Excluded from analysis of withdrawal blood tests	Crohn's disease	Ulcerative colitis/IBDU
**Sustained remission only**		No	125 (86%)	19 (95%)
**Sustained remission plus**	Planning pregnancy	No	5 (3%)	1 (5%)
	Skin problems	No	4 (3%)	0 (0%)
	Other neurological side effect	Yes	2 (1%)	0 (0%)
	Infections	Yes	2 (1%)	0 (0%)
	Transverse myelitis	Yes	1 (1%)	0 (0%)
	Abnormal LFTs	Yes	1 (1%)	0 (0%)
	Anaphylaxis	Yes	1 (1%)	0 (0%)
	Funding	No	1 (1%)	0 (0%)
	Headache	No	1 (1%)	0 (0%)
	Lupus	Yes	1 (1%)	0 (0%)
	Renal cell cancer	Yes	1 (1%)	0 (0%)
	Thrombocytopenia	Yes	1 (1%)	0 (0%)

Supplementary table 2. Characteristics of articles included in systematic review and meta-analysis of withdrawal of anti-TNF for sustained clinical remission, sorted by year of publication.

Abbreviations: IFX infliximab; IMM immunomodulator; CS corticosteroid; CDEIS Crohn's disease endoscopic index of severity; CRP C-reactive protein; hsCRP high-sensitivity C-reactive protein; qPCR quantitative polymerase chain reaction

A: Studies with at least 1 year anti-TNF prior to withdrawal

Study	Methods	Participants	Outcome measure	Outcomes	Predictive factors
**Domenèch 2005**[1]	Retrospective uncontrolled cohort study	27 CD patients (13 luminal, 14 perianal disease) with 1 year IFX maintenance stopped in clinical remission	Clinical relapse	Overall relapse rate: 11/23<br>1-year relapse rate: luminal 17%, perianal 66%, overall 31%	• Perianal disease (p=0.001)
**Wynands 2008**[2]	Retrospective uncontrolled cohort study	11 paediatric CD patients stopped in clinical remission after 12 months' maintenance IFX	Clinical relapse	1-year relapse rate 72% (8/11)	• Not discussed
**Louis 2012**[3]	Prospective uncontrolled cohort study	115 CD patients with at ≥1y combination therapy with IFX and IMM stopped in clinical remission	Clinical relapse	Overall relapse rate: 52/115<br>1-year relapse rate: 43.9 ± 5%<br>2-year relapse rate: 52.2 ± 2%	• CS use 6-12 months pre-withdrawal (p=0.03)<br>• No previous surgical resection (p=0.01)<br>• Male sex (p<0.001)<br>• Haemoglobin < 145 g/L (p<0.001)<br>• Leukocyte count > 6×10^9/L (p=0.01)<br>• CDEIS > 0 (p=0.04)<br>• hsCRP ≥ 5 mg/L (p<0.001)<br>• Infliximab trough level ≥2ml/L (p=0.02)<br>• Faecal calprotectin ≥ 300 µg/g (p=0.04)<br>• In a separate abstract[4], Rajca et al. demonstrate dysbioisis as assessed by qPCR as predictive of relapse.

**Farkas 2013**[5]	Prospective uncontrolled cohort study	51 UC patients with 1 year of anti-TNF therapy stopped in clinical remission	Restart of anti-TNF therapy for clinical relapse	1 year relapse rate: 35%	• Previous biological therapy (p=0.021)
**Molnár 2013**[6]	Prospective uncontrolled cohort study	121 CD patients with 1 year of anti-TNF therapy stopped in clinical remission	Restart of anti-TNF therapy for clinical relapse	1-year relapse rate: 45%	• Smoking (p=0.027)<br>• Corticosteroids at start of anti-TNF (p=0.005)<br>• Previous biological therapy (p=0.013)<br>• Elevated CRP at start of anti-TNF (p=0.025)<br>• Dose-intensification during therapy (p=0.001)
**Chauvin 2014**[7]	Retrospective uncontrolled cohort study	38 CD patients with at ≥1y of combination therapy with IFX and IMM stopped in clinical remission	Clinical relapse or reintroduction of therapy	1-year relapse rate: 44%<br>2-year relapse rate: 64%	Assessed in a combined cohort that included 54 patients who had induction only.<br>• Active smoking (p=0.02)<br>• Previous antimetabolite failure (p=0.03)<br>• Perianal disease (p=0.04)
**Dart 2014**[8]	Retrospective uncontrolled cohort study	9 CD patients with ≥1y anti-TNF therapy stopped in clinical remission	Clinical relapse	1-year relapse rate: 33%	• Not assessed, though scores from STORI study described.
**Molander 2014**[9]	Prospective uncontrolled cohort study	17 CD, 35 UC/IBDU patients with ≥1y anti-TNF therapy stopped in endoscopic and clinical remission; ≥least 6m CS-free remission	Clinical relapse	1-year relapse rate: 29% CD<br>35% UC	• No risk factors on univariable analysis identified
**Brooks 2014**[10]	Prospective uncontrolled cohort study	86 CD patients with ≥ 1 year anti-TNF therapy stopped in clinical remission	Clinical relapse	3-month relapse rate: 4.7%<br>6-month relapse rate: 18.6%<br>1-year relapse rate: 36%	• Ileocolonic disease at diagnosis<br>• Previous anti-TNF therapy

**Monterubbianesi 2015**[11]	Retrospective uncontrolled cohort study	58 CD patients with ≥ 1y anti-TNF therapy stopped in clinical remission	Clinical relapse	1-year relapse rate: 31%<br>2-year relapse rate: 48%<br>5-year relapse rate: 65%	• Mucosal healing not predictive
**Bortlik 2016**[12]	Prospective uncontrolled study	17 UC patients withdrawn from anti-TNF after 14–47m on therapy	Clinical relapse	6-month relapse rate: 23%<br>1-year relapse rate: 23%<br>2-year relapse rate: 36%	• None noted

B: Studies with maintenance therapy but <1 year anti-TNF at withdrawal

Study	Methods	Participants	Outcome measure	Outcomes	Predictive factors
**Waugh 2010**[13]	Retrospective uncontrolled cohort study	48 CD patients with median 15.6m IFX (minimum 1m) withdrawn in clinical remission	Clinical relapse	50% relapse by 477 days<br>Est. 1y relapse rate 44%<br>Est. 2y relapse rate 57%<br>(estimated from Kaplan-Meier curve)	• None identified on multivariable analysis. Genetic markers including *NOD2* polymorphisms and markers in the IBD5 locus were analysed in a a separate paper[14] and were not associated with relapse.
**Nuti 2010**[15]	Prospective uncontrolled study	13 paediatric CD patients and 3 paediatric UC patients stopped in clinical remission	Clinical relapse	1y relapse rate CD 46% (6/13)<br>UC relapses not reported	• None reported
**Steenholdt 2012**[16]	Retrospective uncontrolled cohort study	22 CD patients with median 6 IFX infusions withdrawn in clinical remission<br>(Remaining patients in study induction only or episodic treatment)	Clinical relapse	1-year relapse rate: 39%	• In overall CD cohort, longer disease duration associated with increased risk of relapse.
**Echarri 2013**[17]	Cohort study (unclear if prospective	32 CD patients withdrawn from IFX for deep remission after unknown time on IFX	Clinical relapse	1-year relapse rate: 23%<br>2-year relapse rate: 48%	• Ileal disease, high ASCA levels and faecal calprotectin > 250 µg/g significantly associated with relapse.

	or retrospective)				
**Bortlik 2016**[12]	Prospective uncontrolled cohort study	61 CD patients withdrawn from anti-TNF for steroid-free clinical remission after 4-73 months on therapy	Clinical relapse	6-month relapse rate: 18%<br>1-year relapse rate: 41%<br>2-year relapse rate: 49%	• On multivariable analysisColonic disease (p=0.03) and perianal disease as the indication for anti-TNF (p=0.02) reduced risk.<br>• The presence of perianal disease otherwise increased risk (p=0.02).<br>• No association with biomarker levels (faecal calprotectin, CRP).

C: Studies included in secondary meta-analysis but excluded from primary meta-analysis for other reasons

Study	Methods	Participants	Outcome measure	Outcomes	Predictive factors
**Dai 2014**[18]	Prospective uncontrolled cohort study	109 CD patients, 107 UC patients withdrawn from IFX after 1 year for maintained clinical remission	Relapse or retreatment	1-year relapse rate: 25% CD<br>18% UC<br>(The relapse rates reported in the paper failed to account for the approx. 10% loss-to-follow-up. The rates reported here were estimated based on the number reported to have relapsed within 12 months, rates of loss to follow-up and median/interquartile range of time to relapse; since these rates were estimated, they were excluded from the primary meta-analysis).	• No predictive factors were identified on univariable analysis.

Supplementary table 3: Studies excluded from meta-analysis

Study	Details	Reason for exclusion
**Caviglia 2007**[19]	Retrospective cohort study including 9 CD patients withdrawn from IFX for 'personal reasons'. All still in remission after median 9m follow-up.	No report of 1y relapse rate; unclear whether patients in clinical remission at withdrawal
**Guidi 2008**[20]	Prospective cohort study including 6 patients with perianal CD withdrawn from IFX after a mean of 9.2 infusions. Recurrence in 1/6 patients after 20 months, with successful retreatment. Remainder in remission after mean of 19.4 months.	No report of 1y relapse rate.
**Schnitzler 2009**[21]	Retrospective cohort study including 200 CD patients stopping IFX, but only 12 of these were for reasons other than loss of response or adverse effects.	Most cases had episodic therapy only; no clear cohort stopped for sustained clinical remission; no relapse rate reported for those stopping for other reasons.
**Armuzzi 2010**[22]	Retrospective cohort study including 65 CD patients withdrawn from IFX for sustained clinical remission. 59% remained in remission at median follow-up 16m.	Abstract only; no report of 1y relapse rate
**Armuzzi 2010**[23]	Retrospective cohort study including 69 CD patients withdrawn from IFX for sustained clinical remission.	Abstract only; no separation of patients by reason for drug withdrawal (included patients withdrawn for adverse effects and loss of response also).
**Sorrentino 2010**[24]	Prospective cohort study of 12 CD patients treated immediately after surgery with infliximab, with clinical and endoscopic remission at 24 months and a total of 36 months of therapy prior to withdrawal. Endoscopic recurrence seen at 4 months in 10 of 12 patients, all of whom responded to reduced dose (3 mg/kg) infliximab when reintroduced.	Post-surgical cohort; primary endpoint not clinical recurrence.
**Crombé 2011**[25]	Prospective cohort study including 27 patients with childhood-onset CD who stopped IFX whilst in remission after a median of 16 months' treatment. 23/27 remained in remission after a median follow-up of 125 months.	No report of 1y relapse rate; also insufficient data on reasons for stopping drug.

**Zelinkova 2012**[26]	Prospective study including 12 patients with IBD (subtype not specified) who were withdrawn from adalimumab while in remission during second trimester of pregnancy and then restarting therapy post-partum. Relapses observed in 2 patients (17%)	Abstract only; pregnancy cohort; anti-TNF resumed post-partum even without relapse
**Lee 2013**[27]	Retrospective cohort study including 6 CD patients withdrawn from IFX for remission	Lack of data on time for follow-up
**Luppino 2013**[28]	Retrospective cohort study including 21 CD and 10 UC patients withdrawn from IFX for clinical remission after at least 1 year therapy. 42% relapse rate with median time to relapse of 545 days	Abstract only; no report of 1y relapse rate
**Rismo 2013**[29]	Prospective cohort study of 37 CD patients treated with anti-TNF until endoscopic healing and then withdrawn. Crude relapse rates (not estimated from survival analysis) were 52% at 6 months and 74% at 1 year. Significantly higher mucosal expression of *IL17A* and *TNF* in those who subsequently relapsed than those that did not by Cox proportional hazards.	Included patients who received induction only; no report of 1y relapse rate using survival analysis.
**Sofi 2013**[30]	Meta-analysis of relapse rates after discontinuation of anti-TNF in patients with CD in remission. 398 patients in 4 studies included, with average relapse rates over 1, 2, 3, 4 and 6 years of 35%, 48%, 62%, 69% and 76%.	Abstract only; existing meta-analysis
**Viazis 2013**[31]	Retrospective study including 5 patients with pouchitis treated with IFX for 1 year and then withdrawn after clinical response. All had sustained benefit out to 3 years post-discontinuation.	Pouchitis study
**Buhl 2014**[32]	Prospective double-blind randomised clinical trial of IFX withdrawal in patients with sustained clinical remission	Trial protocol only published at this stage; trial ongoing.
**Zucchi 2014**[33]	Retrospective study including 8 CD and 2 UC patients withdrawn from IFX for deep remission. Relapse rate 75% CD, 0% UC with mean time to relapse of 14 months	Abstract only; no report of 1y relapse rate

**Papamichail 2014**[34]	Retrospective cohort study of 100 CD patients withdrawn from anti-TNF in clinical remission, 65 of which were treated with episodic therapy. Relapse rates 4% at 1y, 7% at 2y, 12% at 3y, 20% at 4y, 27% at 5y. Further abstract from same group[35] identified amphiregulin as predictive of relapse in patients with luminal disease.	Majority of patients did not receive maintenance therapy; no separate reporting of episodic and maintenance groups
**Regueiro 2014**[36]	Prospective study including 8 patients who stopped infliximab after 1 year of post-operative therapy. All 8 had endoscopic recurrence between 8 and 30 months post-cessation (mean of 18.2 ± 7.2 months), and 5 required surgery.	Post-operative study; endoscopic rather than clinical recurrence.
**Muñoz Villafranca 2014**[37]	Prospective study including 12 UC patients with mucosal healing following 54 weeks IFX. 6/12 had new episodes of UC. 3 were retreated; all responded.	Abstract only; no report of 1y relapse rate.
**Ramos 2014**[38]	Retrospective study including 25 CD patients discontinued from anti-TNF for clinical remission. 4/25 (16%) relapse after mean follow-up of 19 months.	Abstract only; no report of 1y relapse rate, nor of time on anti-TNF.
**Ciria 2014**[39]	Retrospective study of 24 CD and 11 UC patients discontinued from anti-TNF for clinical remission greater than 1 year or endoscopic mucosal heaing. Clinical recurrence 35% after median follow-up of 20 months.	Abstract only; no report of 1y relapse rate. No risk factors identified.
**Wolf 2015**[40]	Prospective study comparing 2 matched cohorts of patients with various immune-mediated diseases (including CD and UC) with response to at ≥6m therapy who continued or discontinued therapy. 377 patients in each group. Higher risk of flares in those discontinuing thrapy.	Abstract only; no separation of IBD patients from other disease groups; no report of 1y relapse rate
**Casanova 2015**[41]	Retrospective cohort study including 311 IBD patients (68% CD) withdrawn from anti-TNF for clinical remission. Estimated relapse rates 4% at 6m, 24% at 1y, 37% at 2y, 40% at 3y, 53% at 5y. Higher relapse risk in patients on no immunomodulator on multivariable analysis.	Abstract only; no separation of CD and UC cohorts
**Gisbert 2015**[42]	Systematic review and meta-analysis of risk of relapse after withdrawal from anti-TNF after achieving remission. Overall risk of relapse in CD 44%, in UC 41%. Risk of relapse in CD 36% at 6m, 49% over 60-125m.	Existing meta-analysis; no new data

References for Supplementary Tables

1. Domènech E, Hinojosa J, Nos P, Garcia-Planella E, Cabré E, Bernal I, et al. Clinical evolution of luminal and perianal Crohn’s disease after inducing remission with infliximab: How long should patients be treated? Aliment Pharmacol Ther 2005;22:1107–13.

2. Wynands J, Belbouab R, Candon S, Talbotec C, Mougenot J-F, Chatenoud L, et al. 12-month follow-up after successful infliximab therapy in pediatric crohn disease. J Pediatr Gastroenterol Nutr 2008;46:293–8.

3. Louis E, Mary J-Y, Vernier-Massouille G, Grimaud J-C, Bouhnik Y, Laharie D, et al. Maintenance of remission among patients with Crohn’s disease on antimetabolite therapy after infliximab therapy is stopped. Gastroenterology 2012;142:63–70.e5; quiz e31.

4. Rajca S, Grondin V, Louis E, Vernier-Massouille G, Grimaud J-C, Bouhnik Y, et al. Crohn’s Disease Associated Dysbiosis as a Predictive Factor of Clinical Relapse: A Microbiological Substudy of the GETAID-STORI Cohort. Gastroenterology 2011;140. Epub ahead of print 2011. Available from doi:10.1016/S0016-5085(11)60191-1.

5. Farkas K, Lakatos PL, Nagy F, Szepes Z, Miheller P, Papp M, et al. Predictors of relapse in patients with ulcerative colitis in remission after one-year of infliximab therapy. Scand J Gastroenterol 2013;48:1394–8.

6. Molnár T, Lakatos PL, Farkas K, Nagy F, Szepes Z, Miheller P, et al. Predictors of relapse in patients with Crohn’s disease in remission after 1 year of biological therapy. Aliment Pharmacol Ther 2013;37:225–33.

7. Chauvin A, Le Thuaut A, Belhassan M, Le Baleur Y, Mesli F, Bastuji-Garin S, et al. Infliximab as a bridge to remission maintained by antimetabolite therapy in Crohn’s disease: A retrospective study. Dig Liver Dis 2014;46:695–700.

8. Dart RJ, Griffin N, Taylor K, Duncan J, Sastrillo M, Sanderson J, et al. Reassessment of Crohn’ s disease treated with at least 12 months of anti-TNF therapy: How likely is treatment withdrawal? Frontline Gastroenterol 2014;5:176–82.

9. Molander P, Färkkilä M, Salminen K, Kemppainen H, Blomster T, Koskela R, et al. Outcome After Discontinuation of TNFα-blocking Therapy in Patients with Inflammatory Bowel Disease in Deep Remission. Inflamm Bowel Dis 2014;20:1021–8.

10. Brooks AJ, Sebastian S, Cross SS, Robinson K, Warren L, Wright A, et al. Outcome of elective withdrawal of anti-tumour necrosis factor-α therapy in patients with Crohn’s disease in established remission. J Crohns Colitis 2014; Epub ahead of print 2014. Available from doi:10.1016/j.crohns.2014.09.007.

11. Monterubbianesi R, Papi C, Kohn A. P529. Maintenance of clinical remission in Crohn’s disease patients after discontinuation of antiTNF agents: Results from a single centre cohort. J Crohn’s Colitis 2015;9:S345–S345.

12. Bortlik M, Duricova D, Machkova N, Hruba V, Lukas M, Mitrova K, et al. Discontinuation of anti-tumor necrosis factor therapy in inflammatory bowel disease patients: a prospective observation. Scand J Gastroenterol 2016;51:196–202.

13. Waugh AWG, Garg S, Matic K, Gramlich L, Wong C, Sadowski DC, et al. Maintenance of clinical benefit in

Crohn’s disease patients after discontinuation of infliximab: long-term follow-up of a single centre cohort. Aliment Pharmacol Ther 2010;32:1129–34.

14. Lu C, Waugh A, Bailey RJ, Cherry R, Dieleman L a., Gramlich L, et al. Crohn’s disease genotypes of patients in remission vs relapses after infliximab discontinuation. World J Gastroenterol 2012;18:5058–64.

15. Nuti F, Conte F, Cavallari N, Civitelli F, Aloi M, Del Giudice E, et al. Long term efficacy of infliximab in inflammatory bowel disease at a single tertiary center. J Pediatr Gastroenterol Nutr 2010;50:E108.

16. Steenholdt C, Molazahi A, Ainsworth MA, Brynskov J, Østergaard Thomsen O, Seidelin JB. Outcome after discontinuation of infliximab in patients with inflammatory bowel disease in clinical remission: an observational Danish single center study. Scand J Gastroenterol 2012;47:518–27.

17. Echarri A, Ollero V, Rodriguez JA, Gallego JC, Castro J. Predictors of relapse after discontinuing anti-TNF therapy in Crohn’s disease patients on deep remission. J Crohns Colitis 2013;7:S171.

18. Dai C, Liu W-X, Jiang M, Sun M-J. Mucosal Healing Did Not Predict Sustained Clinical Remission in Patients with IBD after Discontinuation of One-Year Infliximab Therapy. PLoS One 2014;9:e110797.

19. Caviglia R, Ribolsi M, Rizzi M, Emerenziani S, Annunziata ML, Cicala M. Maintenance of remission with infliximab in inflammatory bowel disease: efficacy and safety long-term follow-up. World J Gastroenterol 2007;13:5238–44.

20. Guidi L, Ratto C, Semeraro S, Roberto I, De Vitis I, Papa A, et al. Combined therapy with infliximab and seton drainage for perianal fistulizing Crohn’s disease with anal endosonographic monitoring: a single-centre experience. Tech Coloproctol 2008;12:111–7.

21. Schnitzler F, Fidder H, Ferrante M, Noman M, Arijs I, Van Assche G, et al. Long-term outcome of treatment with infliximab in 614 patients with Crohn’s disease: results from a single-centre cohort. Gut 2009;58:492–500.

22. Armuzzi A, Marzo M, Felice C, Mocci G, De Vincentis F, Andrisani G, et al. OC.07.5 LONG-TERM SCHEDULED THERAPY WITH INFLIXIMAB IN INFLAMMATORY BOWEL DISEASE. Dig. Liver Dis.2010;42:S88.

23. Armuzzi A, Rizzi M, Monterubbianesi R, Marzo M, Cicala M, Guidi L, et al. P.91 THE COURSE OF INFLIXIMAB DISCONTINUATION AFTER LONG-TERM MAINTENANCE THERAPY IN CROHN’S DISEASE. Dig. Liver Dis.2010;42:S135.

24. Sorrentino D, Paviotti A, Terrosu G, Avellini C, Geraci M, Zarifi D. Low-Dose Maintenance Therapy With Infliximab Prevents Postsurgical Recurrence of Crohn’s Disease. Clin Gastroenterol Hepatol 2010;8:591–9.e1.

25. Crombé V, Salleron J, Savoye G, Dupas J-L, Vernier-Massouille G, Lerebours E, et al. Long-term outcome of treatment with infliximab in pediatric-onset Crohn’s disease: A population-based study. Inflamm Bowel Dis 2011;17:2144–52.

26. Zelinkova Z, van der Ent C, Kuipers EJ, van der Woude CJ. Prospective Assessment of the Adalimumab Discontinuation During Pregnancy in Inflammatory Bowel Disease Patients. Gastroenterology 2012;142:S385–

6.

27. Lee L, Boyd J, Lanzon-Miller S. Withdrawal of Maintainence Infliximab Therapy. Gut 2013;62:A34–A34.

28. Luppino I, Spagnuolo R, Marasco R, Cosco C, Ruggiero G, Cosco V, et al. Withdrawal of infliximab (IFX) after achieving remission: Outcome in a cohort of inflammatory bowel disease (IBD) patients. Dig Liver Dis 2013;45:S100–1.

29. Rismo R, Olsen T, Cui G, Paulssen EJ, Christiansen I, Johnsen K, et al. Normalization of mucosal cytokine gene expression levels predicts long-term remission after discontinuation of anti-TNF therapy in Crohn's disease. Scand J Gastroenterol 2013;48:311–9.

30. Sofi AA, Georgescu C, Sodeman T, Nawras A. Physician outlook toward fecal microbiota transplantation in the treatment of Clostridium difficile infection. Am J Gastroenterol 2013;108:1661–2.

31. Viazis N, Giakoumis M, Koukouratos T, Anastasiou J, Katopodi K, Kechagias G, et al. Long term benefit of one year infliximab administration for the treatment of chronic refractory pouchitis. J Crohn's Colitis 2013;7:e457–60.

32. Buhl SS, Steenholdt C, Brynskov J, Thomsen OO, Bendtzen K, Ainsworth MA. Discontinuation of infliximab therapy in patients with Crohn's disease in sustained complete remission (the STOP IT study): protocol for a double-blind, randomised, placebo-controlled, multicentre trial. BMJ Open 2014;4:e005887–e005887.

33. Zucchi E, Fabbro M, Pinese E, Marino M, Panos J, Bulajic M, et al. OC.13.3 OUTCOME OF INFLIXIMAB DISCONTINUATION IN IBD PATIENTS AND THERAPY RECHALLENGING IN RELAPSERS: SINGLE CENTRE PRELIMINARY DATA. Dig Liver Dis 2014;46:S31.

34. Papamichael K, Casteele N Vande, Gils A, Tops S, Hauenstein S, Singh S, et al. Long-Term Outcome of Patients With Crohn's Disease Who Discontinued Infliximab Therapy Upon Clinical Remission. Clin Gastroenterol Hepatol 2014;1–8.

35. Papamichail K, Claes K, de Bruyn M, Hauenstein S, Princen F, Singh S, et al. P431. Serology panel for prediction relapse after discontinuation of infliximab in patients with Crohn's disease achieving clinical remission. J Crohn's Colitis 2015;9:S295–6.

36. Regueiro M, Kip KE, Baidoo L, Swoger JM, Schraut W. Postoperative therapy with infliximab prevents long-term crohn's disease recurrence. Clin. Gastroenterol. Hepatol.2014;12:1494–502.e1.

37. Muñoz Villafranca C, Bravo Rodríguez MT, Ortiz de Zárate J, Arreba González P, García Kamiruaga I, Heras Martín J, et al. P405 Mucosal healing in patients with ulcerative colitis treated with infliximab. What happens after treatment is discontinued? J Crohn's Colitis 2014;8:S234–5.

38. Ramos L, Hernandez Camba a., Carrillo Palau M, Alonso I, Hernandez Alvarez-Buylla N, Quintero Carrion E. P398 Outcome of treatment with biological agents in Crohn's disease: 117 patients in 5 years from a tertiary referral center. J Crohn's Colitis 2014;8:S231.

39. Ciria V, Silva P, Leo E, Trigo C, De la Cruz MD, Herrera JM, et al. P487 Factors influencing recurrence following

suspension of biological treatment. Importance of mucosal healing. J Crohn's Colitis 2014;8:S270.

40. Wolf D, Skup M, Yang H, Faust E, Kageleiry A, Chao J, et al. P504. Clinical outcomes associated with switching or discontinuation of anti-TNF inhibitors for non-medical reasons. J Crohn's Colitis 2015;9:S331–S331.

41. Casanova MJ, Chaparro M, García-Sánchez V, Nantes O, Jáuregui-Amezaga A, Rojas-Feria M, et al. P500. Evolution after anti-TNF drug discontinuation in patients with inflammatory bowel disease (IBD): a multicenter long-term follow-up study. J Crohn's Colitis 2015;9:S329–S329.

42. Gisbert JP, Marín AC, Chaparro M. P472. Risk of relapse after anti-TNF discontinuation in Inflammatory Bowel Disease: A meta-analysis. J Crohn's Colitis 2015;9:S315–S315.

Supplementary Figure 1 – Forest plot for relapse by 12 months after anti-TNF withdrawal for CD (A) and UC/IBDU (B) including all studies with patients treated with maintenance anti-TNF

A

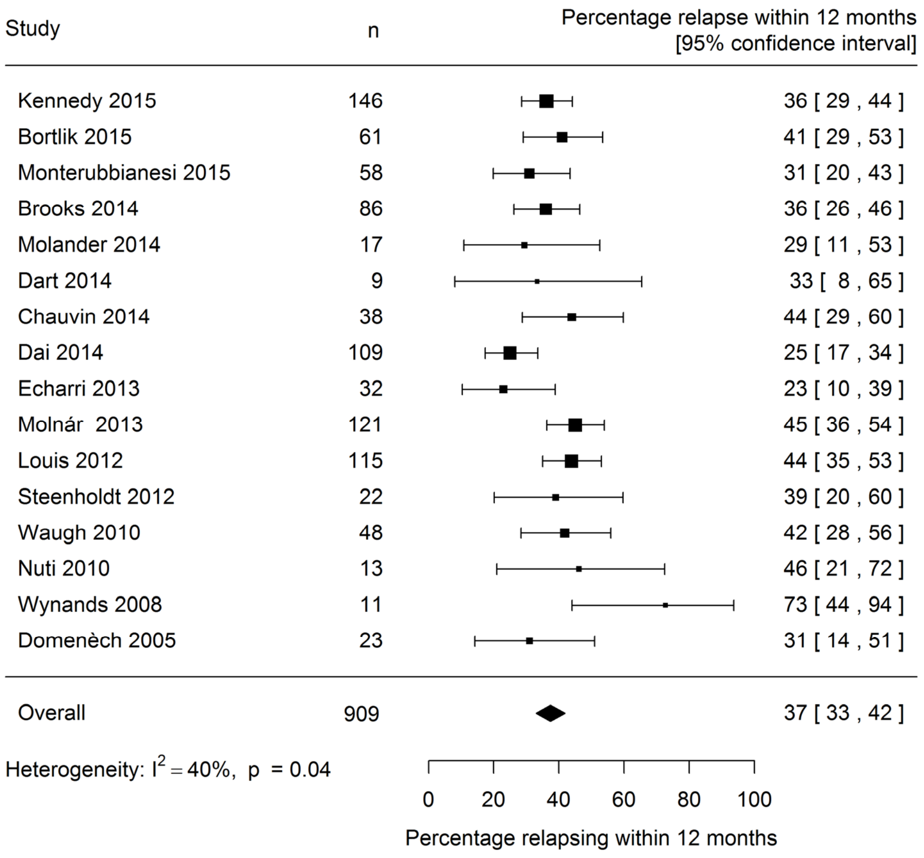

B

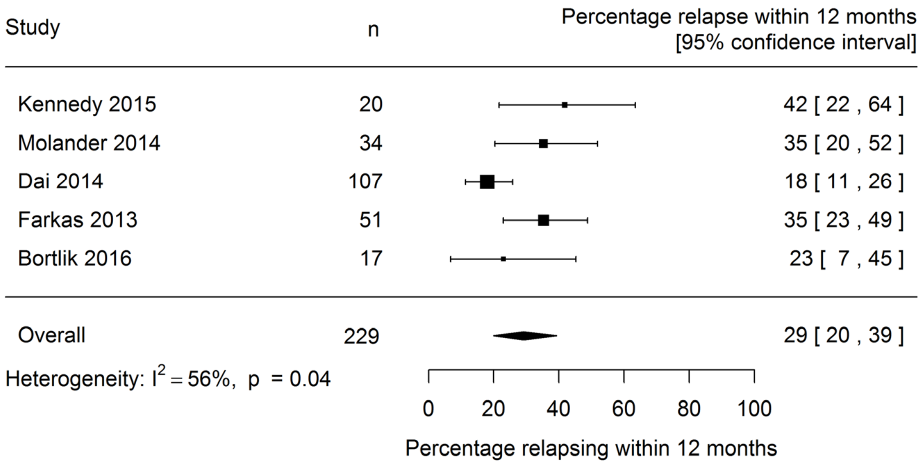

Supplementary Figure 2 – Forest plot for relapse by 24 months after anti-TNF withdrawal for CD (A) and UC/IBDU (B) including only studies with patients treated with maintenance anti-TNF for at least 12 months

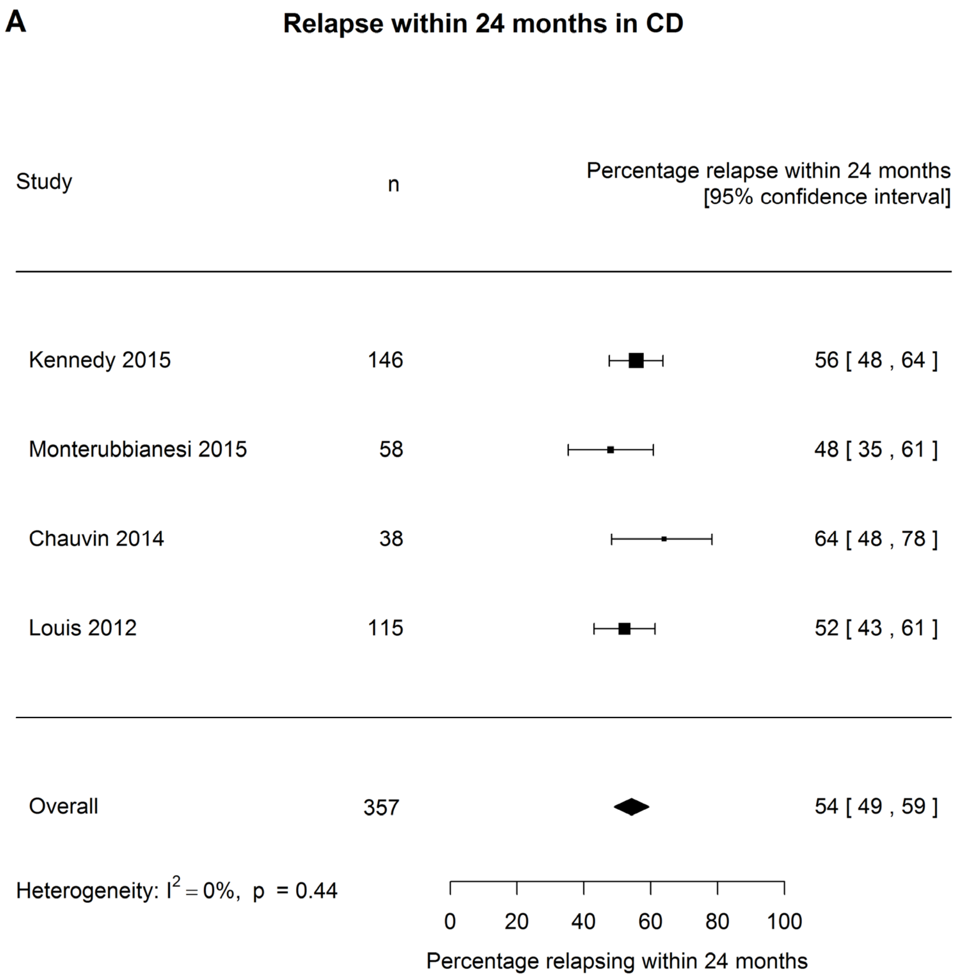

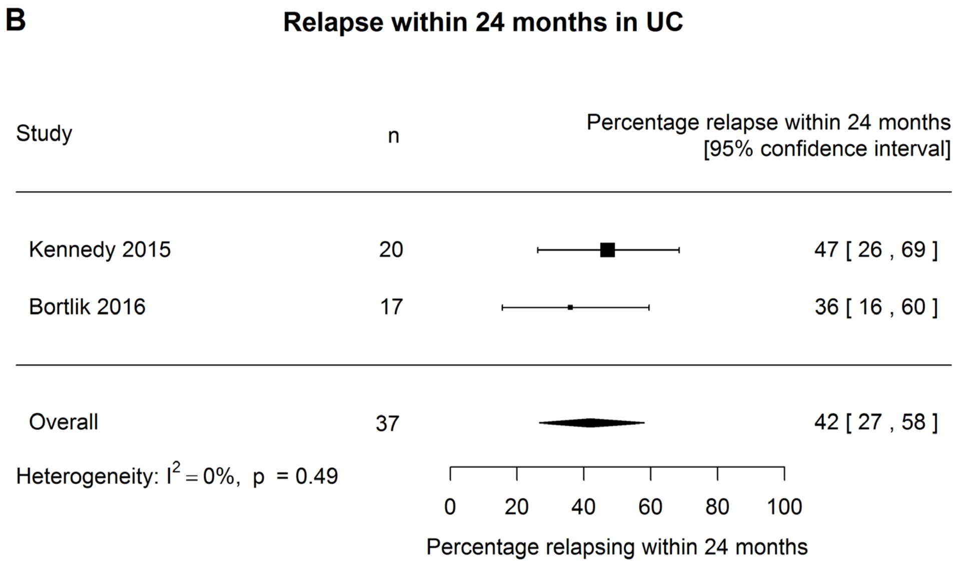

Supplementary Figure 3 – Forest plot for relapse by 24 months after anti-TNF withdrawal for CD including all studies of patients treated with maintenance anti-TNF

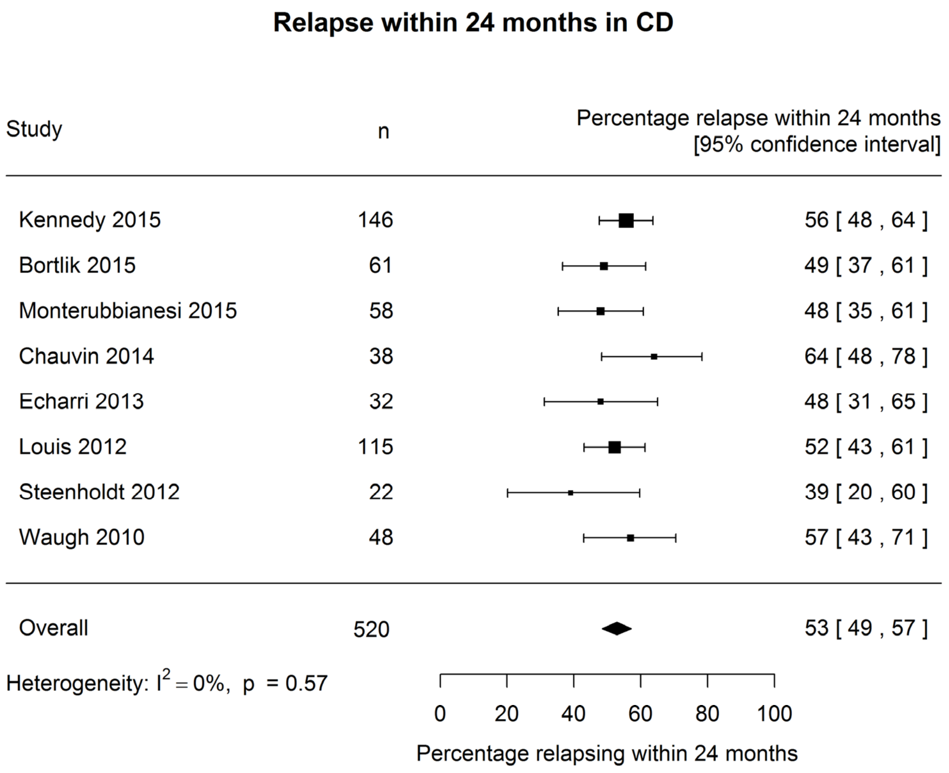

Supplementary Figure 4 – Forest plot for success rates of reintroduction of anti-TNF after withdrawal for CD (A) and UC/IBDU (B) including all studies with patients treated with maintenance anti-TNF

A

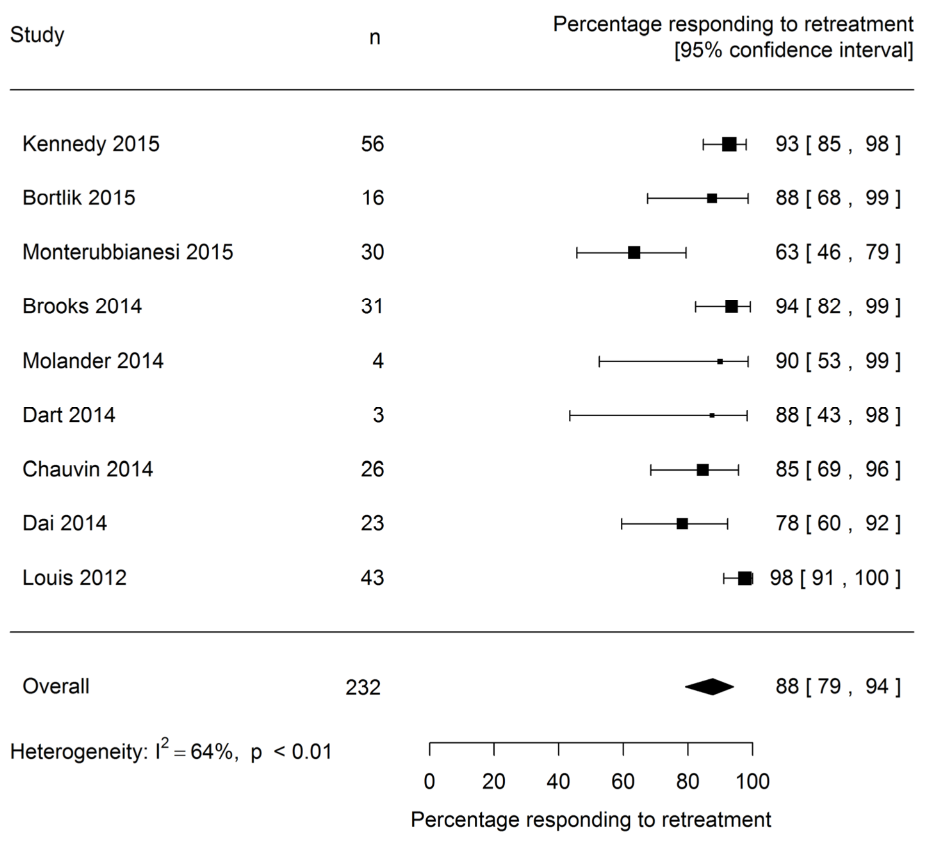

B

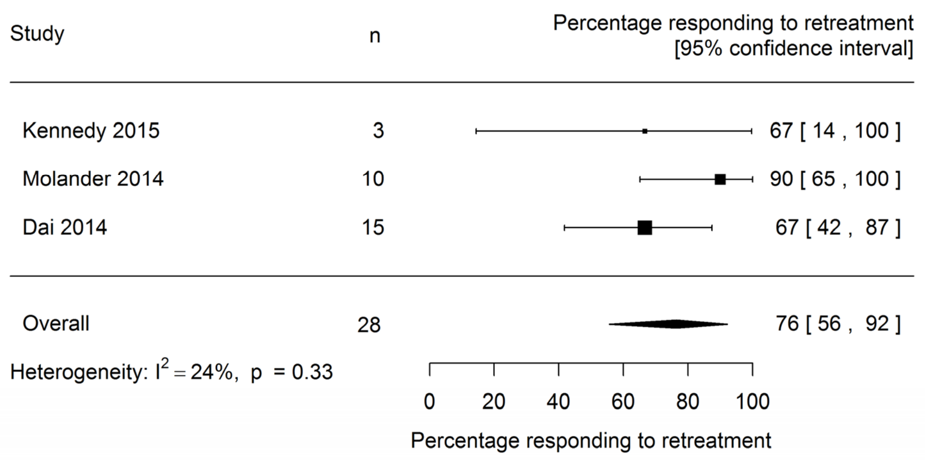

Supplementary Figure 5 – Funnel plot for relapse by 12 months after anti-TNF withdrawal including studies with patients treated with maintenance anti-TNF for at least one year prior to withdrawal

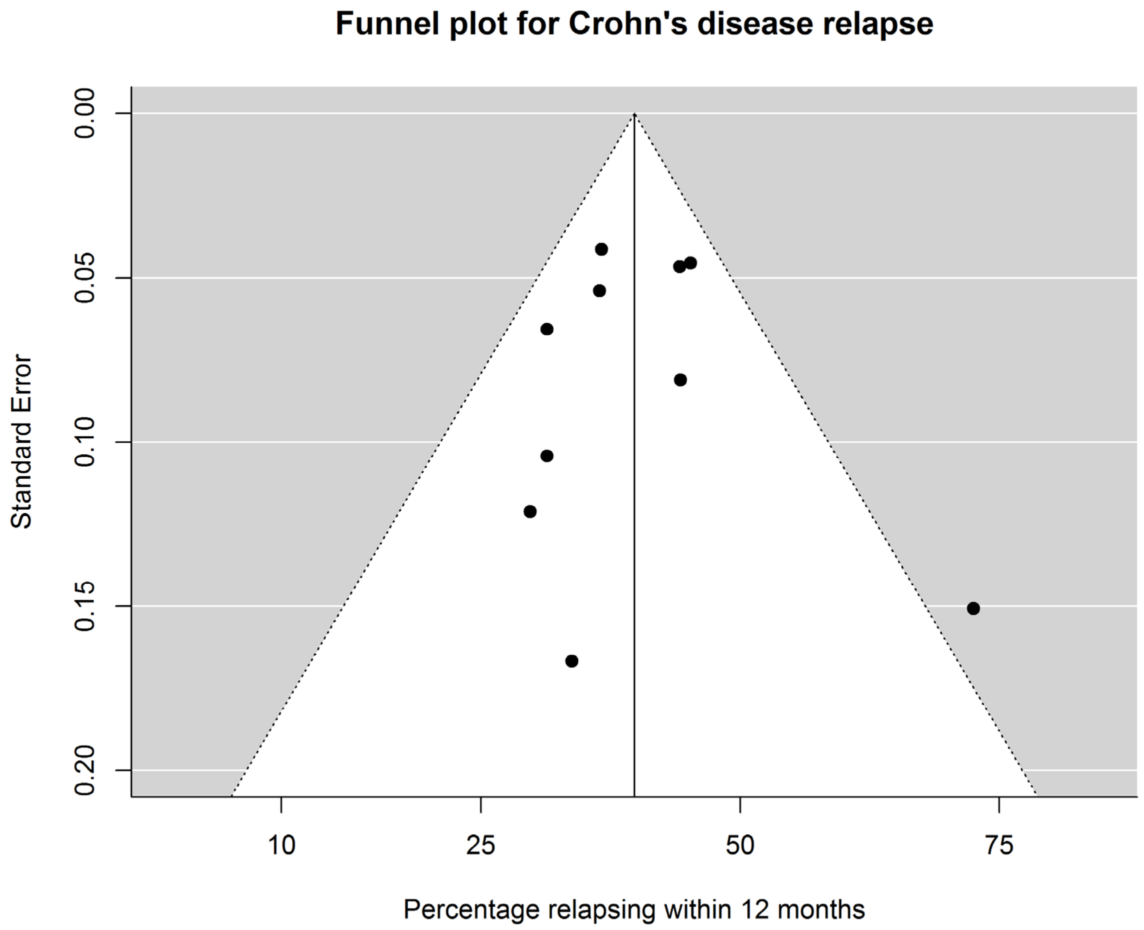

Supplement: Supplementary file 1 — Table S1. Detailed reasons for withdrawal from anti‐TNF. Table S2. Characteristics of articles included in systematic review and meta‐analysis of withdrawal of anti‐TNF for sustained clinical remission, sorted by year of publication. (A) Studies with at least 1 year anti‐TNF prior to withdrawal. (B) Studies with maintenance therapy but <1 year anti‐TNF at withdrawal. C: Studies included in secondary meta‐analysis but excluded from primary meta‐analysis for other reasons. Table S3. Studies excluded from meta‐analysis. Figure S1. Forest plot for relapse by 12 months after anti‐TNF withdrawal for CD (A) and UC/IBDU (B) including all studies with patients treated with maintenance anti‐TNF. Figure S2. Forest plot for relapse by 24 months after anti‐TNF withdrawal for CD (A) and UC/IBDU (B) including only studies with patients treated with maintenance anti‐TNF for at least 12 months. Figure S3. Forest plot for relapse by 24 months after anti‐TNF withdrawal for CD including all studies of patients treated with maintenance anti‐TNF. Figure S4. Forest plot for success rates of reintroduction of anti‐TNF after withdrawal for CD (A) and UC/IBDU (B) including all studies with patients treated with maintenance anti‐TNF. Figure S5. Funnel plot for relapse by 12 months after anti‐TNF withdrawal including studies with patients treated with maintenance anti‐TNF for at least one year prior to withdrawal. [file APT-43-910-s001.pdf]
